# Inhibition of STAT3 Reduces Astrocytoma Cell Invasion and Constitutive Activation of STAT3 Predicts Poor Prognosis in Human Astrocytoma

**DOI:** 10.1371/journal.pone.0084723

**Published:** 2013-12-30

**Authors:** Qinchuan Liang, Chenkai Ma, Yang Zhao, Guodong Gao, Jie Ma

**Affiliations:** 1 Department of Pediatric Neurosurgery, Xin-Hua Hospital, Shanghai JiaoTong University, School of Medicine, Shanghai, People’s Republic of China; 2 Department of Neurosurgery, Tang-Du Hospital, Institution for Functional Neurosurgery of P.L.A., Fourth Military Medical University, Xi’an, Shannxi Province, People’s Republic of China; Beijing Tiantan Hospital, Capital Medical University, China

## Abstract

Astrocytoma cells characteristically possess high invasion potentials. Recent studies have revealed that knockdown of signal transducers and activators of transcription 3 (STAT3) expression by RNAi induces apoptosis in astrocytoma cell. Nevertheless, the distinct roles of STAT3 in astrocytoma’s invasion and recurrence have not been elucidated. In this study, we silenced STAT3 using Small interfering RNAs in two human glioblastoma multiforme (GBM) cell lines (U251 and U87), and investigated the effect on GBM cell adhesion and invasion. Our results demonstrate that disruption of STAT3 inhibits GBM cell’s adhesion and invasion. Knockdown of STAT3 significantly increased E-cadherin but decreased N-cadherin, vascular endothelial growth factor, matrix metalloproteinase 2 and matrix metalloproteinase 9. Additionally, expression of pSTAT3^Tyr705^ correlates with astrocytoma WHO classification, Karnofsky performance status scale score, tumor recurrence and survival. Furthmore, pSTAT3^Tyr705^ is a significant prognostic factor in astrocytoma. In conclusion, STAT3 may affect astrocytoma invasion, expression of pSTAT3^Tyr705^ is a significant prognostic factor in tumor recurrence and overall survival in astrocytoma patients. Therefore, STAT3 may provide a potential target for molecular therapy in human astrocytoma, and pSTAT3^Tyr705^could be an important biomarker for astrocytoma prognosis.

## Introduction

Astrocytoma is the most common primary tumors of the brain. Based on degree of malignancy, astrocytoma is graded into grade ii (diffuse astrocytoma), grade iii (anaplastic astrocytoma), and grade iv (glioblastoma multiforme, GBM) [1-5]. GBM is the most common type with the worst prognosis, and average post-operative survival is less than 2 years [6]. Astrocytoma cells characteristically possess high proliferation and invasion potentials, which explain their aggressive phenotype [7]. In this regard, elucidating the molecular mechanism of cell motility and invasion in astrocytoma is important for the development of more effective treatment options in the future.

 Signal transducers and activators of transcription (STAT) is a family of transcription factors and is involved in a wide variety of cellular physiological processes, including differentiation, survival, or cell growth [8]. Recently, increasing evidence has suggested that abnormalities in STATs signaling, especially STAT3, are involved in the oncogenesis of several cancers [6,9,10]. For example, constitutive activation of STAT3 correlates with cell proliferation in breast [11] and non-small-cell lung carcinoma [10]. However, there is little knowledge of the relationship between astrocytoma and STATs. Recent studies have revealed that knockdown of STAT3 expression by RNAi induces apoptosis in astrocytoma cells [12,13]. Nevertheless, thus far, the distinct roles of STAT3 in astrocytoma’s invasion and recurrence have not been elucidated, nor have the downstream targets been evaluated. 

 Astrocytoma cells, possessing high invasion potentials, are unique in their striking property to migrate and invade normal brain. These features are not shared with other cancer cells that are often metastatic to the other organs. In the present work, we assess the biological significance of STAT3 in astrocytoma invasion, and evaluate the relationship with astrocytoma prognosis. We silenced STAT3 using siRNA in two human glioblastoma multiforme cell lines (U251 and U87), and investigated the effect on cell adhesion and invasion. We also evaluated the changes in expression of several proteins that directly relate to cell adhesion and invasion. In addition, STAT3 expression and activation were quantified using immunohistochemistry in human astrocytoma samples to determine the relationship with prognosis and clinical outcome. The overall aim of this study was to determine the precise roles of STAT3 in human astrocytoma progression, and to test the hypothesis that STAT3 signaling may be a novel predictor of astrocytoma prognosis and a potential therapeutic target.

## Materials and Methods

### Ethics Statement

Archival specimens were obtained with informed consent from 78 astrocytoma patients at Tang-Du Hospital. All patients provided written informed consent before enrollment, and the study protocol was approved by the Ethic Committee of Fourth Military Medical University, and the study was carried out according to the provisions of the Helsinki Declaration of 1983.

### Cell culture, Small interfering RNA and Cell transfection

Two human GBM cell lines (U251 and U87) were cultured in DMEM medium (Gibco, Shanghai, China), supplemented with 10% fetal bovine serum (FBS) at 37°C in a humidified 5% CO2 atmosphere. The cell lines U251 and U87 used in this study were reserved in our lab. We had ever investigated the role of STAT5a/5b isoforms in human glioblastoma multiforme (GBM) progression using these U251 and U87 cells. And we had ever reported the study in Cancer Letters in 2009 [6].

 Small interfering RNAs (siRNAs) were obtained from Dharmacon Inc. (USA) and used to target human STAT3 (GenBank accession number NM_003150). Twenty-four hours before transfection at 30–40% confluence, cells were transferred to 6-well plates. Transfection of siRNAs was carried out with DharmaFECT 1 siRNA transfection reagent (Dharmacon Inc, USA) according to the manufacturer’s instructions. Cells were collected for analysis 48 h after transfection. Nonspecific siRNA (Dharmacon Inc.) was used as a negative control and the selective silencing of STAT3 was confirmed by Western blotting.

### Western Blotting and Antibodies

Whole-cell lysates were prepared from cancer cell lines, and Western blot analysis was performed using standard techniques as described previously. Proteins were detected using the enhanced chemiluminescence detection kit (SuperSignal West Femto Substrate, Pierce). Glyceraldehyde-3-phosphate dehydrogenase (Kangchen, Shanghai, China) was detected as a loading control. Antibodies used in this study were purchased from Cell Signaling Technology Inc. (USA). All primary antibodies were used at a 1:1000 dilution.

### ELISA Analysis of VEGF

Cells (1×10^5^) respectively transfected with STAT3 or control siRNA (50 nM) were maintained in serum-free medium for 48 h. The medium was collected, and the concentrations of vascular endothelial growth factor (VEGF) in the medium were determined using an enzyme-linked immunosorbent assay (ELISA) kit (R&D systems, USA) according to the manufacturer’s instruction.

### Gelatin Zymography

To check the expression and activation of matrix metalloproteinase (MMP), cells were transfected with STAT3 or control siRNA (50 nM) in serum-free medium for 48 h. The conditioned media were collected by centrifugation, concentrated, and dialyzed. The dialyzed samples containing an equal amount of total proteins were mixed with sample buffer, incubated in a water bath (55°C) for about 3 to 5 mins, and separated by 8% acrylamide gels containing 0.1% gelatin. The gels were incubated in 2.5% Triton X-100 solution at room temperature with gentle agitation and then were soaked in reaction buffer [50 mM Tris-HCl (pH 7.5), 200 mM NaCl, 10 mM CaCl_2_] at 37°C overnight. Afterwards, gel was stained with 0.25 % coomassie blue in 50 % methanol and 10 % acetic acid for 4 h, and then destained for 0.5 h. MMPs were detected as clear band against a blue background.

### Cell Adhesion Assay

A 96-well culture plate was coated with Matrigel (BD Bioscience, Franklin Lakes, NJ, USA) and blocked with PBS containing 2% BSA, then washed with PBS. Cells in serum-free medium containing 0.1% BSA were added to the wells (2 × 10^4^ cells/well) and incubated at 37°C for 60 min. After removing the medium and nonattached cells, 0.1% crystal violet was added for 10 min. The plate was gently washed with tap water and dried in air for 24 h. Then 0.1ml of 5% SDS with 50% ethanol was added for 20 min and the absorbance was read at 540 nm.

### In Vitro Invasion Assay

Cell invasion assays were performed as described by Hecht et al. [14]. In brief, chambers with 8-μm-pore polycarbonate membranes, coated with Matrigel on the upper side, were used (Becton Dickinson, San Diego, CA). Cells were transfected with siRNA (50 nM) for 48 h. And then cells (1 × 10^5^) in 250 μl of serum-free medium were seeded into the upper chamber, whereas medium supplemented with 15 % fetal bovine serum was applied to the lower chamber as a chemo-attractant to induce invasion. Cells transfected with nonspecific siRNA were used as the negative control. After incubation for 24 h, nonmigrated cells on the upper chamber of the filter were removed with a cotton swab. Migrated cells on the bottom surface of the filter were fixed, stained and counted.

### Astrocytoma Cases and Follow-up

Archival specimens were obtained with informed consent from 78 astrocytoma patients at Tang-Du Hospital (Xi’an, People’s Republic of China) between December 2007 and June 2009. None of the patients had received preoperative anti-tumor treatment. Clinical features including age, gender, Karnofsky performance status scale (KPS) score and WHO classification (high-grade gliomas: grade III/IV; low-grade gliomas: grade I/II) were obtained from the medical records. Patients were followed-up until June 30, 2011, with a mean follow-up of 12.9 months (range: 1–30 months). Patients who died due to causes unrelated to tumors or without a complete follow-up prior to death were excluded from the present study.

### Immunohistochemical Staining

The tissue sections were deparaffinized in xylene and rehydrated with a graded series of ethanol. A three-step streptavidin-biotin-horseradish peroxidase method was used, and STAT3, pSTAT3^Tyr705^ were assessed with primary antibodies against STAT3 (1:100) and pSTAT3^Tyr705^ (1:50) using the LSAB+ kit (DakoCytomation, Denmark) according to the manufacturer’s instructions.

 The slides were examined independently by two investigators blinded to both the clinical and pathological data. Protein expression was quantified based on the extent of staining (percentage of positive tumor cells) and classified into two categories: negative (0-15% tumor cells positive) and positive (more than 15% tumor cells positive) [15-17].

### Statistical Analysis

Statistical analysis was performed using SPSS 13.0 (SPSS, Chicago, IL, USA), and the value of *P* < 0.05 was assigned to be statistically significant. Results were expressed as the mean±SD. The Student’s *t* test was used for comparisons between groups. The associations between two categorical variables were evaluated using the Chi-square test. Overall survival (OS) was defined as the interval between surgery and death or the last follow-up. Progression-free survival (PFS) was defined as the time from initial surgical diagnosis to tumor progression in MRI or death from glioma. The Kaplan-Meier method was used to determine survival probability and differences were assessed by the log-rank test. Cox’s proportional hazards regression model was used for analysis of independent prognostic factors. 

## Results

### Disruption of STAT3 Inhibits Adhesion and Invasion of Glioblastoma Multiforme Cells

**Figure 1:**
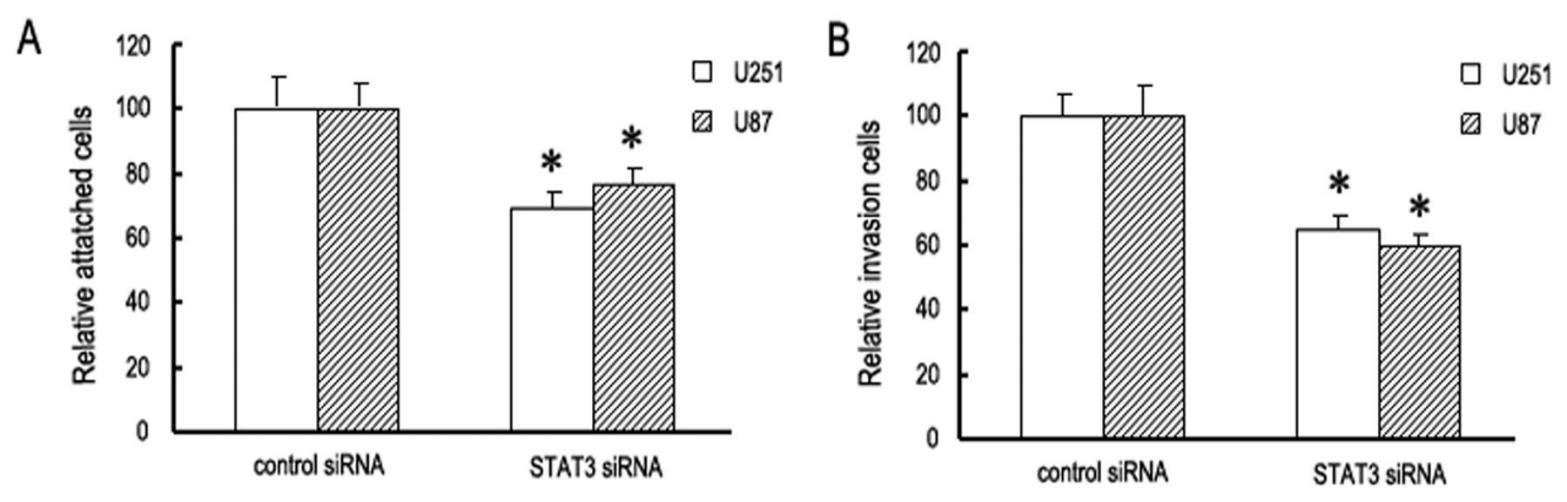
The functional roles of STAT3 in GBM cells adhesion and invasion. Cell adhesion assay (A) and cell invasion assays (B) were adopted. At 48 h post-transfection, STAT3 siRNA suppressed GBM cells adhesion and invasion. Data are shown as mean ± SD from six separate experiments. (*P < 0 .05 compared with control siRNA group).

Adhesion and invasion are important processes in tumor invasion. We first addressed the functional roles of STAT3 in GBM cells adhesion and invasion. In the cell adhesion assay, 48 h after STAT3 siRNA transfection, attached cells were significantly decreased in both U251 and U87 cells (Figure 1A). In the invasion assay, knockdown of STAT3 expression significantly reduced the invasion ability of GBM cells. For instance, 48 hours after transfection with 50 nM of STAT3 siRNA, the number of U251 and U87 cells able to migrated through the filter decreased to 65.3% and 59.5%, respectively, when compared to cells transfected with nonspecific siRNA (Figure 1B). These results indicate that STAT3 may have significant effects on adhesion and invasion in GBM cells.

### Decreased STAT3 Activation is Associated with Modulation of E-cadherin, N-cadherin, VEGF, MMP2 and MMP9

**Figure 2:**
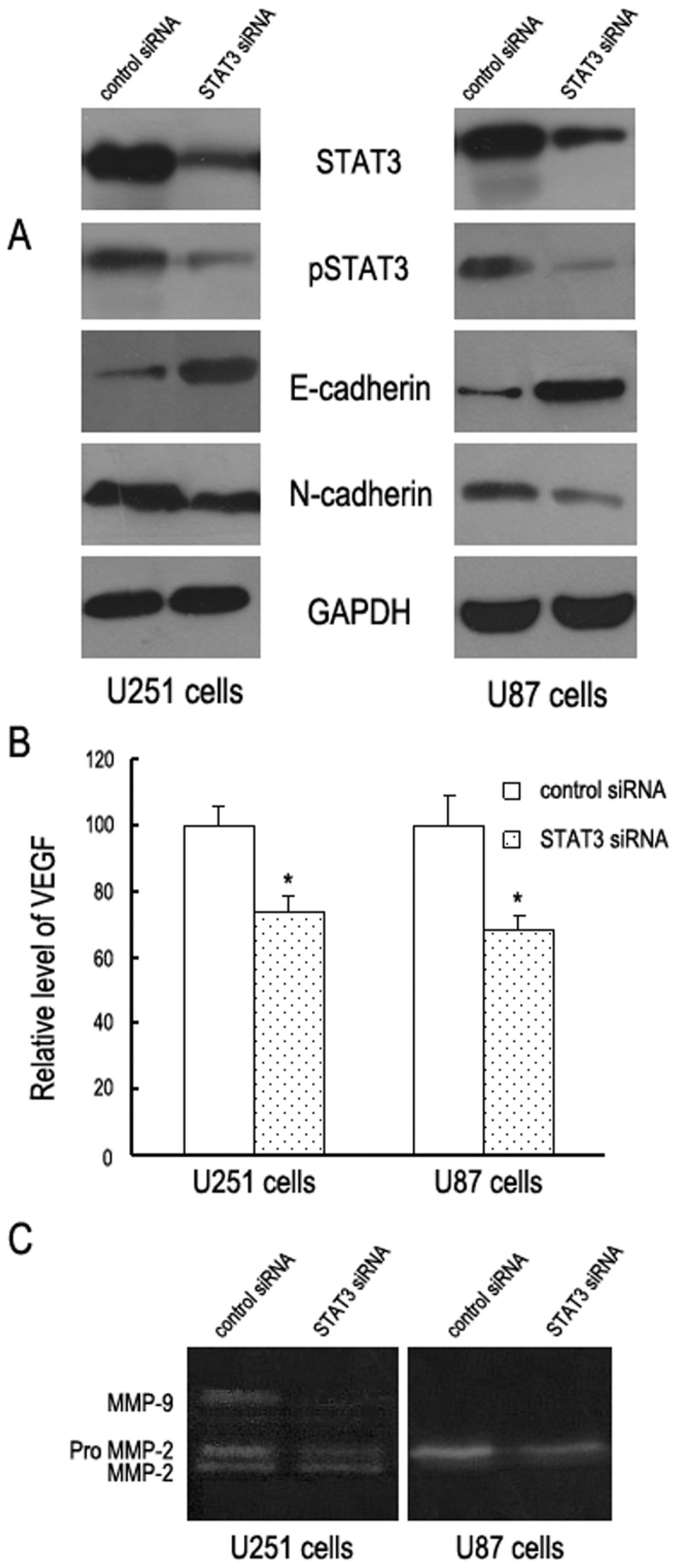
Decreased STAT3 activation is associated with modulation of E-cadherin, N-cadherin, VEGF, MMP2 and MMP9. A representative Western blot (A), ELISA analyses (B) and gelatin zymography (C) showed that knockdown of STAT3 expression significantly decreased the phophorylation of STAT3 and the expressions of STAT3, N-cadherin, VEGF, MMP2 and MMP9, while E-cadherin expression was dramatically increased in both U251and U87 cells, indicating that STAT3 may participate in the regulation of invasiveness and metastasis in human astrocytoma.

To better understand the mechanisms of STAT3 signaling on GBM cells invasion, we examined the expression of various migration and invasion regulatory proteins by Western blot and ELISA analyses. Our data indicated that, knockdown of STAT3 significantly increased E-cadherin, but decreased N-cadherin (Figure 2A) and VEGF expressions (Figure 2B). In addition, the results of gelatin zymography showed that MMP2 and MMP9 secretions were significantly decreased in STAT3 siRNA transfected GBM cells, when compared to nonspecific siRNA transfected cells. (Figure 2C).

### Activated STAT3 Correlates with Advanced Clinico-pathological Features in Human Astrocytoma

A total of 78 patients were recruited for this study; 10 cases were withdrawn due to loss during follow up (n = 4), death due to other causes (n = 3) and poor slide quality (n = 3). In the remaining population (n = 68) of 43 males and 25 females, the mean age was 40.3 years (range: 10–64 years).

**Figure 3:**
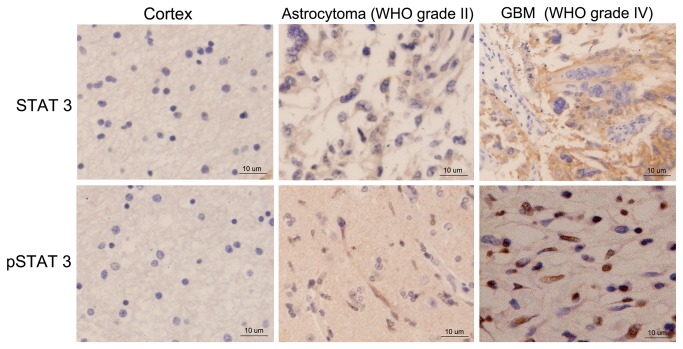
Immunohistochemical staining of human astrocytoma tissue. STAT3 expression is detected in the cytoplasm. pSTAT3^Tyr705^ expression is mainly observed in the nucleus, magnification 200×.

STAT3 is activated through the phosphorylation of tyrosine at residue 705. Thus, pSTAT3^Tyr705^ is an essential protein for the activation of STAT3 signaling. Immunohistochemical staining reveals that STAT3 staining was detected mainly in the cytoplasm, while phosphorylated STAT3 (pSTAT3^Tyr705^) expression was mostly detected in the nucleus (Figure 3). As shown in table 1, In STAT3 positive and negative patients, there was no significant difference in gender, age, WHO classification and KPS score. However, pSTAT3^Tyr705^ was detected in 7/28 (25.0%) of the low-grade I/II specimens and 27/40 (67.5%) of the high-grade III/IV specimens, indicating that positive pSTAT3^Tyr705^ was significantly more common in high-grade astrocytoma tissues. Additionally, a significant relationship was also observed between pSTAT3^Tyr705^ expression and the KPS score. Our data showed that pSTAT3^Tyr705^ occurred more frequently in tumors with low KPS score than those with high KPS score (*P* = 0.003). Taken together, pSTAT3^Tyr705^ levels, but not gender or age, were associated with WHO classification (*P* = 0.001) and KPS score (*P* = 0.003) in human astrocytoma.

**Table 1:** Correlation between STAT3, pSTAT3^Tyr705^ and clinicopathological characteristics in astrocytoma patients.

		STAT3		*P* value	p-STAT3^705^		*P* value
		Positive	Negative		Positive	Negative	
Gender	Male	28	15	0.809	21	22	0.801
	Female	17	8		13	12	
Age	>40	22	10	0.672	18	14	0.331
	<40	23	13		16	20	
WHO classification	I/II	17	11	0.426	7	21	0.001*
	III/IV	28	12		27	13	
KPS score	>80	27	11	0.339	13	25	0.003*
	<80	18	12		21	9	

pSTAT3^Tyr705^ levels are associated with WHO classification (*P* = 0.001) and KPS score (*P* = 0.003), suggesting that constitutive activation of STAT3 may be associated with poorer prognosis in human astrocytoma.

### Expression of Activated STAT3 Correlates with Poorer Prognosis in Human Astrocytoma

**Figure 4:**
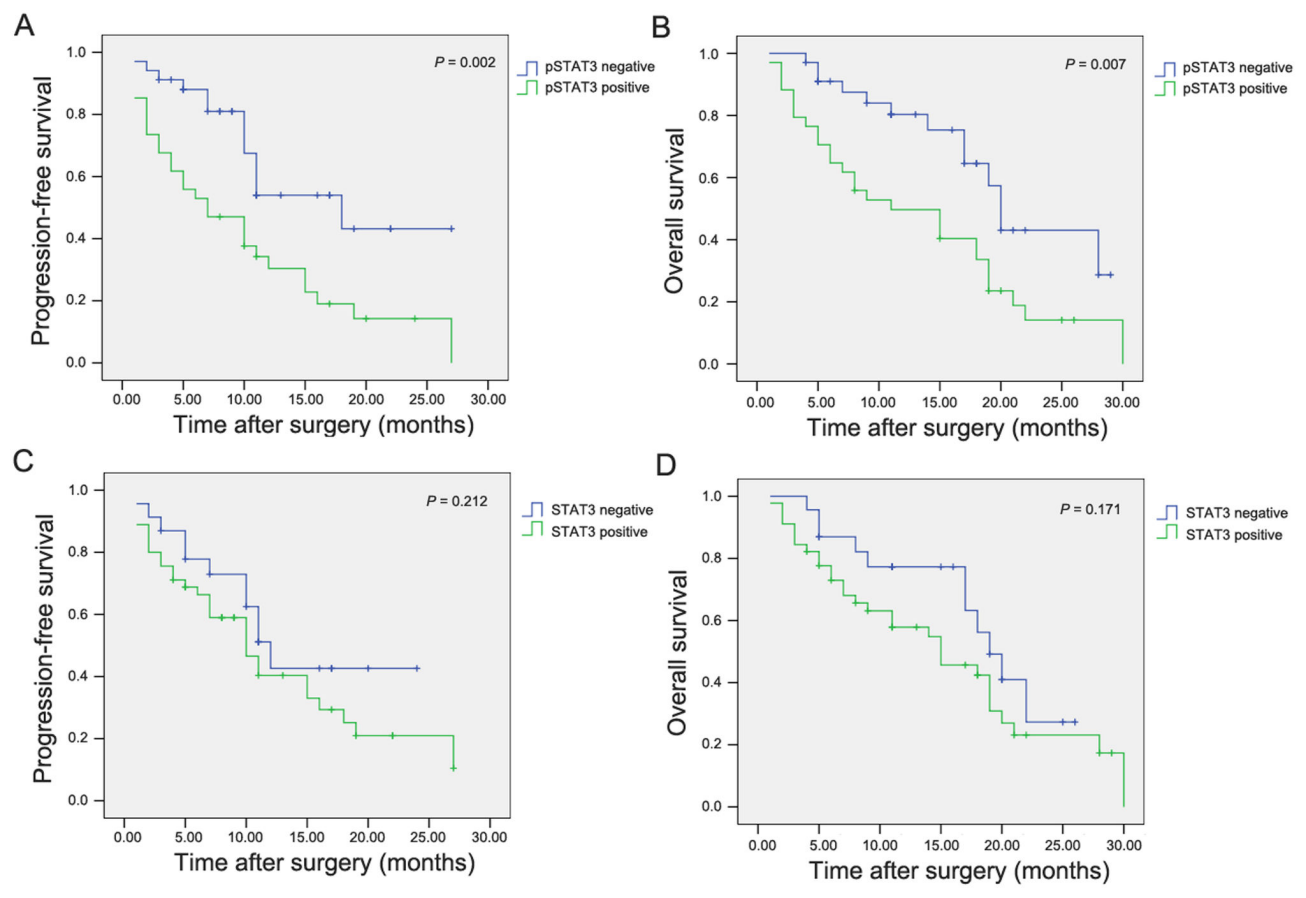
Prognostic significance assessed by Kaplan-Meier analysis and log-rank tests. The astrocytoma patients with positive pSTAT3^Tyr705^ expression have poorer prognosis in terms of (A) progression-free survival (*P* = 0.002) and (B) overall survival (*P* = 0.007). However, the progression-free survival and overall survival in astrocytoma patients were not related to the expression of STAT3 (C and D).

Furthermore, the association of STAT3/pSTAT3^Tyr705^ with prognosis in astrocytoma patients was determined. Patients with positive pSTAT3^Tyr705^ expression had a significantly poorer prognosis than pSTAT3^Tyr705^ negative patients (PFS, *P* = 0.002; OS, P = 0.007; Figure 4A/4B). The 1 and 2-year OS rates for pSTAT3^Tyr705^ positive patients were 47.1% and 8.9%, respectively, versus 78.1% and 40.9%, in pSTAT3^Tyr705^ negative patients. Similarly, the median OS time of pSTAT3^Tyr705^ positive patients (11.0 months) was significantly shorter than pSTAT3^Tyr705^ negative patients (20.0 months). Additionally, the 1 and 2-year PFS rates for pSTAT3^Tyr705^ positive patients were 31.6% and 13.9%, respectively, versus 52.7% and 41.3%, in pSTAT3^Tyr705^ negative patients. However, STAT3 was not associated with differences in PFS and OS (*P* = 0.212 and 0.171, respectively, Figure 4C/4D). 

 The prognostic value of STAT3/pSTAT3^Tyr705^ expression was also evaluated using univariate analysis, which showed that STAT3 expression, age, gender and tumor size had no prognostic significance for PFS and OS. However, the WHO classification and KPS were predictors for PFS and OS. pSTAT3^Tyr705^ expression was also a significant predictor for tumor recurrence and OS in astrocytoma patients (Table 2). Moreover, multivariate analysis was conducted and confirmed that positive pSTAT3^Tyr705^ expression was still the independent variable for predicting recurrence and OS in astrocytoma patients (*P* = 0.013 and *P* < 0.001, respectively).

**Table 2:** Cox’s proportional hazards regression of independent prognostic factors in astrocytoma.

Variables	Progression-free survival			Overall survival		
	Univariate	Multivariate analysis		Univariate	Multivariate analysis	
	(*P* value)	Hazard ratio (95%) CI	*P* value	(*P* value)	Hazard ratio (95%) CI	*P* value
Age, years (<40 versus >40)	0.272			0.321		
Gender (male versus female)	0.315			0.198		
WHO classification (I/II versus III/IV)	<0.001	1.736 (1.121-2.427)	0.032	<0.001	1.936 (1.481-2.623)	0.027
KPS score (>80 versus <80)	<0.001	3.056 (2.236-5.022)	<0.001	<0.001	3.301 (2.216-4.972)	<0.001
STAT3 (positive versus negative)	0.317			0.275		
pSTAT3 (positive versus negative)	<0.001	2.001 (1.509-2.877)	0.013	<0.001	2.301 (1.726-3.021)	<0.001

WHO classification, KPS and pSTAT3Tyr705 expression were independent prognostic factors for both overall survival and disease-free survival, results were considered significant if the P value was less than 0.05.

## Discussion

Despite intensive therapy including surgery followed by radio- and chemotherapy, astrocytoma is still incurable. This is mainly due to the high propensity of astrocytoma cells to invade the surrounding normal brain, which prevents a complete tumor resection [18]. However, the mechanism of metastasis and the factors affecting the metastasis in astrocytoma are not fully understood, which impedes the development of effective treatments.

 STATs are a family of transcription factors which are involved in the regulation of numerous tumor associated genes and act as important cellular mediators in response to various cytokines and growth factors [8,19,20]. Recently, constitutive activation of STAT proteins has been detected frequently in several malignant neoplasms. Also, studies have proposed that STAT3 signaling may be involved in cell viability and apoptosis regulation in astrocytoma carcinogenesis [12,13]. However, the precise roles of STAT3 signaling in human astrocytoma invasion and recurrence have not been fully characterized. In our previously study, we had ever detected the expressions of JAK2 and pJAK2^Tyr1007/Tyr1008^, STAT3’s upstream, in the normal cortex and human astrocytoma tissues using immunohistochemistry. Our data showed that no significant differences in their expressions were seen in both normal cortex and human astrocytoma tissues. In the present study, we first evaluated the biological significance of STAT3 in astrocytoma cells adhesion and invasion. The cell adhesion and Matrigel invasion assay showed that an inverse relationship between the adhesion/invasiveness of GBM cells and inhibition of STAT3 genes. Thus, our results indicated that STAT3 may have significant effects on adhesion and invasion in GBM cells.

 Glioma cells invasion is a complex and multi-step mechanism involving a large array of molecules and cell–cell and cell–extracellular matrix (ECM) interactions [21]. The next step was to elucidate the molecular players involved in the invasion. We examined the expression of various migration and invasion regulatory proteins by Western blot and ELISA analyses. Our data suggest that blockade of STAT3 activity decreases the expression of N-cadherin, MMP2, MMP9 and VEGF, but increases the expression of E-cadherin, suggesting that STAT3 may be involved in the regulation of the expression of E-cadherin, N-cadherin, MMPs, and VEGF. These results imply that STAT3 signaling may regulate multiple processes in astrocytoma cells invasion. First, The loss of E-cadherin expression is a crucial step in the initiation of tumor metastasis and a fundamental event in epithelial-mesenchymal transition [6,22]. Knockdown of STAT3 significantly increased E-cadherin and decreased N-cadherin, implying that STAT3 might contribute to epithelial-mesenchymal transition progression in GBM U251 and U87 cells. Second, invasion of astrocytoma cells in the brain is preceded by a process of proteolysis and solubilization of the ECM that enables tumor cells to spread [23]. STAT3 activation, by stimulating MMP2 and MMP2 production, could induce degradation of the ECM. Third, treatment with STAT3 siRNA reduces VEGF secretion by GBM cells, suggesting that STAT3 could regulate astrocytoma invasive capability by affecting angiogenesis. Thus, for the first time, we provide mechanistic evidence that STAT3 might affect astrocytoma invasion by multiple mechanisms including contribution to epithelial-mesenchymal transition progression, enzymes-based degradation of the ECM, angiogenesis, adhesion and invasion.

 On the other hand, few studies have examined prognostic factors in astrocytoma. To determine whether STAT3 was associated with the clinical outcome of astrocytoma, we examined STAT3 expression and activation in 68 human astrocytoma samples. In this study, there was no significant correlation between total STAT3 expression and clinical characteristics such as WHO classification and KPS score; however, we observed a positive correlation between expression of pSTAT3^Tyr705^ and WHO classification and KPS score, suggesting that activation of STAT3 may contribute to a poor prognosis in human astrocytoma. Furthermore, patients with positive pSTAT3^Tyr705^ expression had a significantly poorer prognosis than pSTAT3^Tyr705^ negative patients. The 1 and 2-year OS rates for pSTAT3^Tyr705^ positive patients were lower. The median OS time of pSTAT3^Tyr705^ positive patients was significantly shorter than pSTAT3^Tyr705^ negative patients. Moreover, most importantly, patients with pSTAT3^Tyr705^-positive tumors had an increasing risk of recurrence, since a significantly reduced progression-free survival disease rate were showed in this study. Additionally, univariate and multivariate analyses revealed that pSTAT3^Tyr705^ expression is a powerful independent predictor for tumor recurrence and OS in astrocytoma patients.

## Conclusion

 In summary, our data demonstrate that STAT3 may affect astrocytoma invasion, expression of pSTAT3^Tyr705^ is a significant prognostic factor in tumor recurrence and OS in astrocytoma patients. Thus, STAT3 may provide a potential target for molecular therapy in human astrocytoma, and pSTAT3^Tyr705^could be an important biomarker for astrocytoma prognosis. 
